# Performance of an artificial intelligence model compared with multiple human experts in scoring synovitis and osteophyte severity on joint ultrasound images

**DOI:** 10.1016/j.ero.2026.01.015

**Published:** 2026-03-02

**Authors:** Anders Bossel Holst Weber, Lene Terslev, Mads Ammitzbøll-Danielsen, Bill Aplin Frederiksen, Hilde Berner Hammer, Benjamin Schultz Overgaard, Thiusius Rajeeth Savarimuthu, Søren Andreas Just

**Affiliations:** 1 ROPCA ApS, Odense, Denmark; 2 Center for Rheumatology and Spine Disease, Rigshospitalet, Glostrup, Denmark; 3 Section of Rheumatology, Department of Medicine, Svendborg Hospital - Odense University Hospital, Svendborg, Denmark; 4 Center for Treatment of Rheumatic and Musculoskeletal Diseases (REMEDY), Diakonhjemmet Hospital, Oslo, Norway; 5 Faculty of Medicine, University of Oslo, Oslo, Norway; 6 Mærsk Mc-Kinney Møller Institute, University of Southern Denmark, Odense, Denmark

## Abstract

**Objectives:**

*To* evaluate the agreement of an artificial intelligence (AI) model with human expert raters in assessing greyscale synovitis, Doppler activity, and osteophytes in hand joints.

**Methods:**

Ultrasound images of the wrist, metacarpophalangeal, proximal interphalangeal, distal interphalangeal, and interphalangeal joints were collected. Five experienced rheumatologists, all ultrasound instructors, scored images for synovial hypertrophy (SH), Doppler activity, and osteophyte severity on a 0 to 3 scale using established scoring systems. The AI model was trained, validated, and tested on 7314 images, then compared against raters on 1280 images for SH, 840 videos for Doppler, and 351 images for osteophytes. Agreement was calculated as the AI’s average agreement with all raters.

**Results:**

For SH, the AI vs expert raters showed a kappa value of 0.39 (95% CI, 0.35-0.44), a percent exact agreement (PEA) value of 51.77% (95% CI, 48.83-54.70), and a percent close agreement (PCA) value of 91.03% (95% CI, 89.21-92.63). For Doppler activity, the kappa value was 0.61 (95% CI, 0.54-0.67), the PEA value was 80.49% (95% CI, 77.51-83.22), and the PCA value was 97.13% (95% CI, 95.69-98.18). For osteophyte grading, the kappa value was 0.56 (95% CI, 0.48-0.64), the PEA value was 70.69% (95% CI, 65.57-75.45), and the PCA value was 96.28% (95% CI, 93.70-98.01). Interrater reliability among the human experts showed comparable kappa value ranges: 0.36 to 0.47 for SH, 0.69 to 0.74 for Doppler, and 0.42 to 0.64 for osteophytes.

**Conclusions:**

The AI model demonstrated agreement with expert raters comparable with interrater agreement for SH and osteophyte grading, whereas it was slightly lower for Doppler activity. The lower-than-expected human interrater reliability, particularly for SH, may reflect the absence of prereading alignment sessions, which provide a more realistic picture of variability in expert scoring. These findings support the potential of AI-assisted ultrasound interpretation, while underscoring the need for continued model refinement.

**WHAT IS ALREADY KNOWN ON THIS TOPIC**

 

• Artificial intelligence (AI) is rapidly being explored in rheumatology imaging, with several models showing promise in assessing specific ultrasound features like osteophytes. However, most existing AI models focus on a single feature of a single disease, limiting their clinical utility.

**WHAT THIS STUDY ADDS**

 

• This study introduces a novel AI model capable of assessing multiple hallmarks of both inflammatory and degenerative joint diseases (synovitis, Doppler activity, and osteophytes) across different joint types. The model achieved agreement levels comparable to the variability observed among experienced rheumatologists without prior calibration. While promising, further technical refinement is needed to optimise performance across all features and joint types.

**HOW THIS STUDY MIGHT AFFECT RESEARCH, PRACTICE OR POLICY**

 

• This study contributed to the early validation of AI-based tools for musculoskeletal ultrasound analysis and highlighted their potential role in supporting standardised image interpretation. Although the AI model demonstrated performance comparable with interexpert variability in image scoring, further model refinement and prospective clinical evaluation are necessary.

Alt-text: Unlabelled box dummy alt text

## INTRODUCTION

The lack of rheumatologists is an increasing problem worldwide, posing a massive risk for early detection and disease monitoring of patients with destructive inflammatory arthropathies [1]. Rheumatoid arthritis (RA) is the most prevalent of these diseases [2]. Inflammation in one or more of the joints of the hands can be detected in over 99% of patients at the time of RA diagnosis [3]. Accurate and timely assessment of synovitis, particularly in small hand joints, is therefore crucial for early diagnosis and timely intervention. Joint ultrasound imaging has become a tool in the assessment of synovitis, enabling the visualisation of synovial inflammation. As part of the European Alliance of Associations for Rheumatology (EULAR)-Outcome Measures in Rheumatology (OMERACT) scoring system for grading synovitis activity on ultrasound images, both synovial hypertrophy (SH) and Doppler activity are scored from 0 to 3 [4].

Ultrasound imaging is highly sensitive in detecting structural changes such as osteophytes, joint effusion, and joint space narrowing, which are features that may also occur in inflammatory arthritides [5,6]. Discriminating between hand osteoarthritis (OA) and inflammatory joint diseases can therefore be difficult and must be based on an integrated clinical assessment, including patient history, physical examination, and laboratory findings [7]. The Outcome Measures in Rheumatology (OMERACT) hand OA working group has provided an ultrasound scoring system for grading osteophyte severity [5,8]. Although ultrasound can detect osteophytes with high sensitivity, their clinical significance remains uncertain, as they are common with age and are not sufficient to fulfil the American College of Rheumatology (ACR) clinical criteria for hand OA [9]. Currently, no international hand OA definition includes ultrasound-based osteophyte grading, and their interpretation requires clinical context.

In previous studies, we have demonstrated the feasibility of using artificial intelligence (AI) to grade arthritis and osteophytes in OA in separate AI models [[10], [11], [12]].

AI models that assess joint ultrasound images for both inflammatory (synovitis and Doppler) and degenerative (osteophytes) changes may support clinicians in forming a more complete joint assessment when integrated with clinical findings, anamnesis, and laboratory data. Rather than replacing clinical judgement, such models could help standardise ultrasound interpretation, reduce interrater variability, and enable consistent evaluation across larger patient populations.

The aim of this paper was to evaluate the performance of a developed AI algorithm, DIANA, for grading both synovitis (SH and Doppler activity) and osteophytes on ultrasound images of hand joints. The model’s performance is evaluated following the respective EULAR-OMERACT scoring systems by comparing the algorithm against multiple expert rheumatologists.

## METHODS

### AI model design

**Figure 1:**
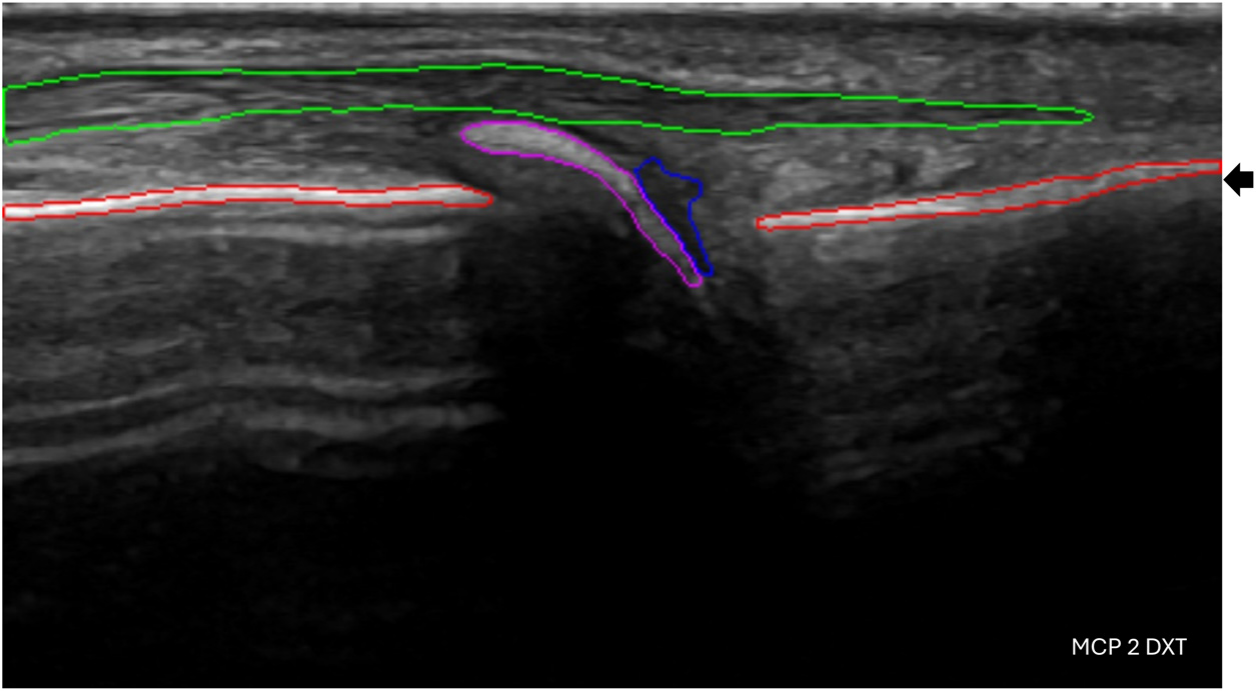
Example output from the AI segmentation and disease activity scoring model (DIANA). The image shows a longitudinal dorsal view of a metacarpophalangeal (MCP) joint. Bone is marked in red, tendon in green, synovium and cartilage combined in blue, and bone with osteophyte formation in purple. The ultrasound machine’s focus point is marked using an arrow.

The AI algorithm, DIANA, is based on a framework previously developed and validated for individual ultrasound features [[10], [11], [12]]. In the current study, these components are integrated into a single AI system utilising a 2-stage sequential workflow (pipeline). In the first stage, a segmentation network (U-Net-based) is employed to outline specific anatomical structures, including a combined region of interest (ROI) for the SH and hyaline cartilage, as well as bones, osteophytes, tendons, and artefacts (Fig 1). This segmentation serves to isolate the clinically relevant area from the surrounding tissues.

In the second stage, a separate classification algorithm analyses the image data specifically within these segmented ROIs to assign a semiquantitative score (0-3). This 2-stage architecture—where segmentation-derived ROIs are used for subsequent disease grading—is consistent with established methodologies in musculoskeletal AI research [[13], [14], [15]]. For a detailed description of the AI design, please see the Supplementary Material. The AI’s classification rules are based on the respective OMERACT guidelines, and detailed descriptions of the grading systems for each pathology are provided in Supplementary Table S1 [8,16].

For osteophyte assessment, the AI model was trained using expert-segmented ultrasound images annotated according to the validated OMERACT semiquantitative scoring system [8]. The severity grade is determined by the vertical displacement from the cortical bone and the largest protrusion, as defined by Hammer et al [8]. These expert-labelled segmentations and scores were used for supervised training of a convolutional neural network (CNN), which was optimised to predict the correct grade based on input image features.

Regarding SH, the model was trained to treat cartilage and synovium as a single anatomical region in the initial segmentation (Fig 1). This is due to the fact that these structures are often indistinguishable on dorsal midline ultrasound images because of the high fluid content of hyaline cartilage. However, the grading of SH is performed by a classification network trained on image-level ground truth scores provided by expert rheumatologists. Since these experts provided scores by accounting for the presence of normal hyaline cartilage, the model learns to map the image features to the correct clinical grade. Consequently, the model’s output reflects the expert-level assessment of SH rather than a simple measurement of the total segmented thickness.

### Training data

The ultrasound data for the AI model were acquired between 2019 and 2024 from clinical studies conducted at the Section of Rheumatology at Svendborg Hospital, Odense University Hospital, Denmark [11,12,17,18]. The patients from these studies consist of both early and established patients with RA and patients referred on the suspicion of inflammatory joint disease, which included hand OA, psoriatic arthritis, and patients without subsequent signs of joint disease on imaging. All patients signed informed consent, and all trials were approved by the Ethical Board, in compliance with Danish law. The use of these images for the development and validation of the AI model, including the most recent expansion to osteophyte grading, was assessed by the Regional and National Ethics Committees (Videnskabsetiske Komitéer for Region Syddanmark and De Videnskabsetiske Medicinske Komitéer). The committees ruled that the existing informed consents were sufficient for this purpose, and no further consent was required, as previously described in Overgaard et al [12].

All ultrasound images were acquired by experienced musculoskeletal sonographers during previous studies. Before model training and testing, all images underwent quality assurance by 1 of 2 experienced sonographers (SAJ or BO). Images were excluded if they lacked sufficient anatomical clarity or were judged to be of suboptimal quality.

A General Electric Logiq 9 or 10 ultrasound scanner was used with an ML6-15 ultrasound probe to obtain the images. The Doppler signal gain was set to a sensitivity just below the disappearance of colour noise. The Doppler frequency was 10.3 MHz, the pulse repetition frequency 0.8, and the wall filter 86 Hz. The data include ultrasound images of metacarpophalangeal (MCP) joints, proximal interphalangeal (PIP) joints, distal interphalangeal (DIP) joints, the interphalangeal (IP) joint of the thumb, and radiocarpal intercarpal (RCIC) joints of the hands, scanned in the longitudinal plane from the dorsal side of the hand.

The AI model was trained, validated, and tested on a total of 7314 images and videos. The specific test sets, where expert raters and the AI model evaluated identical cases, consisted of 1280 static images from 275 subjects for SH, 840 video clips from 47 subjects for Doppler activity, and 351 static images from 140 subjects for osteophytes. This ensured a direct and fair comparison between human assessments and AI predictions across all evaluated features.

All static ultrasound images were acquired in the midline dorsal view of each joint, following EULAR–OMERACT technical recommendations for synovitis scoring [19]. Osteophytes were assessed in the MCP, PIP, IP, and DIP joints. SH and Doppler were assessed in the MCP, PIP, and wrist joints (RCIC). A detailed overview of the number and type of joints assessed for each feature is provided in Supplementary Table S2.

### Patient and public involvement

Patients or the public were not involved in the design, conduct, reporting, or dissemination of this research.

### Study design

#### Expert ultrasound assessments

Five rheumatologists (LT, HBH, SAJ, BF, and MAD), all ultrasound experts and teachers, participated in evaluating and scoring the ultrasound images and videos. The workload was distributed across the 3 pathology domains to ensure multiple human assessments for each case. Three experts (SAJ, LT, and BF) independently scored the full test sets for all 3 domains (1280 SH images, 840 Doppler videos, and 351 osteophyte images). Additionally, HBH scored the full test sets for SH and osteophytes, whereas MAD participated in scoring the SH test set.

Due to the voluntary nature of participation, not all raters completed every domain. To address this, we performed a sensitivity analysis restricted to the raters who completed all SH scorings to confirm the robustness of our results.

The scoring was conducted independently and blinded from the other raters’ and the AI’s assessments using the open-source software CVAT [20]. This platform enabled consistent visualisation and synchronised scoring, reducing potential variation due to differing presentation formats. All scores followed the OMERACT 4-grade (0-3) semiquantitative scales for synovitis and osteophytes [8,16]. Raters had access to and were encouraged to utilise the OMERACT ultrasound atlas during the process to ensure consistent interpretation. Raters were also permitted to skip any image or video if the quality did not meet their standards. No reader alignment sessions or consensus calibration meetings were conducted before scoring.

#### AI-based ultrasound assessments

DIANA was trained using standardised ultrasound images of joints labelled and segmented by expert rheumatologists according to EULAR–OMERACT definitions. DIANA thus automatically identifies and grades pathologies (SH, Doppler, and osteophytes) directly from image data alone, without the need for additional predefined clinical or diagnostic terms.

The segmentation data (7314 ultrasound images) were split into 3 parts: a training set and a validation set, used during training of the segmentation model, and a test set, which was used after all training was completed to evaluate its performance. To avoid bias, data for the evaluation of the disease grading did not overlap with any data used during the training of the segmentation model. For details on the number of images for each joint used in training the AI model, please see Supplementary Table S2. The SH and Doppler test sets included MCP, PIP, and wrist RCIC joints, but not DIP joints. The osteophyte test set included MCP, PIP, IP, and DIP joints, but not wrist RCIC joints. For details on the performance of the trained segmentation network on each respective tissue type in the test data, please see Supplementary Table S3.

### Statistics

Performance metrics, including Cohen’s kappa, percent exact agreement (PEA), percent close agreement (PCA), sensitivity, specificity, positive predictive value (PPV), and negative predictive value (NPV) values, were calculated with 95% confidence intervals (CI). The weighted kappa value was used for ordinal variables (disease activity scores from 0 to 3).

PCA value was calculated as the proportion of scores within ± 1 grade of the reference score. To evaluate the AI model’s ability to detect clinically relevant pathology, we calculated sensitivity, specificity, PPV, and NPV values. Pathological findings were defined as a score of 2 or 3, and normal findings as 0 or 1, using the EULAR–OMERACT scoring thresholds. For SH and Doppler, this binary threshold (0-1 = normal and 2-3 = pathological) follows the consensus-based EULAR–OMERACT ultrasound scoring system [4,16]. For osteophytes, no such consensus definition currently exists. We therefore applied a pragmatic dichotomisation of 0 to 1 as normal and 2 to 3 as pathological, in line with prior exploratory OA ultrasound studies [5,8]. This approach facilitated the calculation of sensitivity, specificity, and binary agreement values but should be interpreted with caution. The majority rating of the expert raters served as the reference standard. These binary metrics were calculated separately for SH, Doppler activity, and osteophyte assessments.

The weighted Cohen’s kappa (ordinal weights) value was calculated and interpreted according to Landis and Koch: < 0.20 poor, 0.21 to 0.40 fair, 0.41 to 0.60 moderate, 0.61 to 0.80 good, and 0.81 to 1.00 very good agreement [21].

For all agreement analyses (AI vs raters and interrater comparisons), only joint images or videos that were scored by both parties were included. Missing data, such as images not scored by a given rater, were excluded on a pairwise basis for each relevant comparison. No imputation was performed. The test set included multiple images from different joints of the same patients. We did not account explicitly for intrapersonal correlation in our analyses, and this should be considered a limitation.

The agreement between raters and AI was calculated as the AI’s average agreement with all human raters. The agreement between the raters was calculated as the average agreement with the other human raters, not including the AI assessment.

The reference standard for calculating binary metrics (sensitivity, specificity, accuracy) was defined by the majority rating of the expert raters. In the event of a tie where no majority could be established, a sensitive approach was adopted by selecting the higher grade as the reference standard to ensure that potential pathological features were not underestimated.

To evaluate the AI model’s performance, we utilised 2 distinct approaches for agreement metrics: (1) comparison against the majority-vote reference standard for binary classification and (2) the average agreement between the AI and each individual rater using weighted Cohen’s kappa values. The latter approach accounts for interrater variability, providing a more robust measure of how the AI performs relative to the distribution of human expert assessments.

A sensitivity analysis was conducted to address the incomplete scoring by some raters. For SH, we repeated the AI-rater and interrater comparisons using only data from readers 1 to 3, who completed all SH assessments.

Data were analysed using Stata version 18.5 from StataCorp.

## RESULTS

The distribution of disease grades by the expert consensus, the AI model, and the human raters is shown in Table 1. The expert consensus, defined by the majority rating of the rheumatologists, served as the reference standard. For SH, 83.7% (1071/1280) of the expert consensus scores indicated either absence (grade 0) or mild presence (grade 1) of pathology. For Doppler activity, 90.7% (762/840) were graded as 0 or 1 by consensus, and for osteophytes, 83.7% (294/351) were similarly low-grade (0 or 1). As shown in the “Expert Consensus” column of Table 1, these figures represent the benchmark for the binary performance metrics.

**Table 1:** Distribution of disease grades by artificial intelligence (AI) and human raters

	Grade	Expert consensus % (n)	AI model % (n)	Rater 1 % (n)	Rater 2 % (n)	Rater 3 % (n)	Rater 4 % (n)	Rater 5 % (n)
SH	0	56.6 (725)	40 (516)	42 (538)	39 (498)	67 (855)	25 (321)	27 (347)
	1	27.0 (346)	43 (553)	38 (486)	37 (475)	13 (171)	39 (493)	34 (437)
	2	11.4 (146)	8 (105)	11 (144)	15 (186)	8 (97)	21 (264)	15 (193)
	3	5.0 (63)	8 (106)	3 (43)	2 (20)	2 (24)	4 (47)	3 (42)
	No grade		0 (0)	5 (69)	8 (101)	10 (133)	12 (155)	20 (261)
Doppler	0	81.5 (685)	77 (643)	77 (646)	73 (617)	79 (664)		
	1	9.2 (77)	14 (118)	6 (53)	14 (121)	5 (46)		
	2	7.1 (60)	7 (60)	4 (33)	6 (52)	3 (29)		
	3	2.1 (18)	2 (19)	2 (17)	1 (12)	0 (3)		
	No grade		0 (0)	11 (91)	5 (38)	12 (98)		
Osteophytes	0	72.1 (253)	70 (246)	70 (244)	84 (294)	56 (195)	60 (209)	
	1	11.7 (41)	21 (74)	15 (52)	14 (49)	28 (99)	28 (99)	
	2	11.1 (39)	6 (22)	8 (27)	1 (4)	10 (35)	7 (25)	
	3	5.1 (18)	3 (9)	5 (16)	0 (1)	3 (11)	3 (9)	
	No grade		0 (0)	3 (12)	1 (3)	3 (11)	3 (9)	

Distribution of disease grades by expert consensus, the AI model, and individual human raters. The expert consensus column represents the majority-vote reference standard used for performance analysis. Ungraded joint images were excluded from calculations. Grey fields indicate that the joint was not scored by that specific rater. SH, synovial hypertrophy.

**Figure 2:**
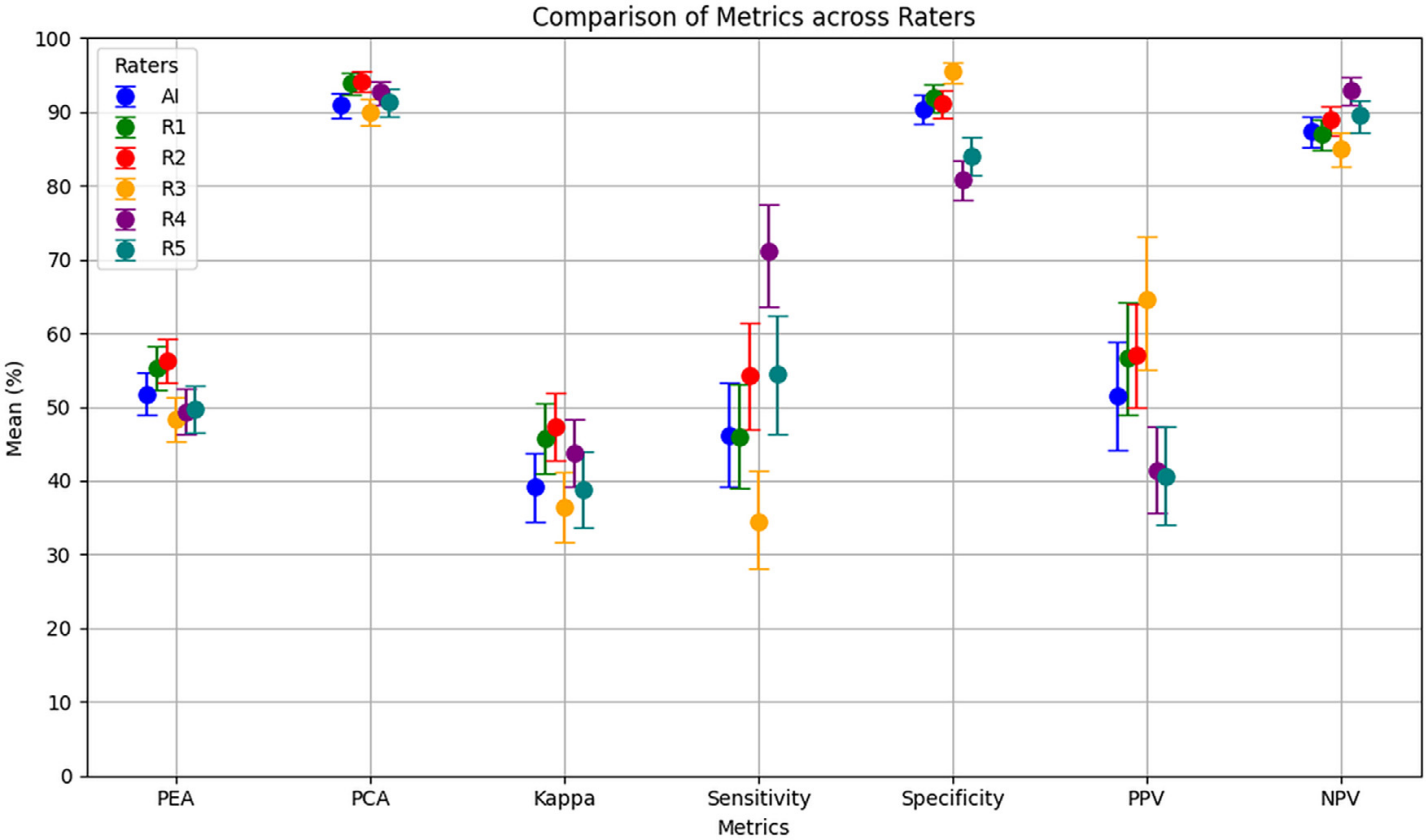
Synovial hypertrophy (SH) agreement. The SH grading agreement of the AI vs all human raters is shown in blue. The performance of individual human raters vs the other human raters is shown as follows: rater 1 (R1) in green, rater 2 (R2) in red, rater 3 (R3) in yellow, rater 4 (R4) in purple, and rater 5 (R5) in dark green. Mean values and 95% CIs are visible in the plot. NPV, negative predictive value; PCA, percent close agreement; PEA, percent exact agreement; PPV, positive predictive value.

The agreement of the AI model to score SH compared with the 5 experts is shown in Figure 2.

For SH, the AI vs raters agreement showed a kappa value of 0.39 (95% CI, 0.35-0.44), a PEA value of 51.77% (95% CI, 48.83-54.70), a PCA value of 91.03% (95% CI, 89.21-92.63), a sensitivity value of 46.19% (95% CI, 39.13-53.32), and a specificity value of 90.43% (95% CI, 88.35-92.25). As can be seen in Figure 2, PEA, PCA, and kappa values for the raters are very similar to those of the AI values. Additional performance metrics, including PPV and NPV, are available in Supplementary Table S4.

**Figure 3:**
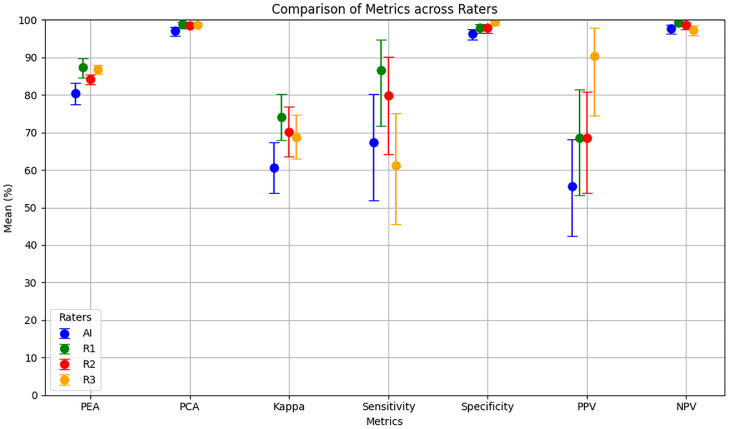
Doppler activity agreement. Doppler activity grading agreement of the AI vs all human raters is shown in blue. The performance of individual human raters vs the other human raters is shown as follows: rater 1 (R1) in green, rater 2 (R2) in red, and rater 3 (R3) in yellow. Mean values and 95% CIs are visible in the plot. NPV, negative predictive value; PCA, percent close agreement; PEA, percent exact agreement; PPV, positive predictive value.

To assess the impact of missing data, a sensitivity analysis was performed, including only readers 1 to 3 (who completed the entire test set). This analysis confirmed the primary findings: the AI vs rater agreement for SH showed a weighted kappa value of 0.39 (95% CI, 0.34-0.44), a PEA value of 53.82% (95% CI, 50.94-56.67), and a PCA value of 91.28% (95% CI, 89.52-92.82). These values, which exclude the partial scores from raters 4 and 5, demonstrate that the AI’s performance remains stable and comparable with the interrater reliability of the most consistent human experts (detailed results in Supplementary Table S5). The agreement of the AI model to score Doppler activity compared with the 3 experts is shown in Figure 3. For Doppler activity the agreement of the AI vs raters had a kappa value of 0.61 (95% CI, 0.54-0.67), a PEA value of 80.49% (95% CI, 77.51-83.22), a PCA value of 97.13% (95% CI, 95.69-98.18), a sensitivity value of 67.31% (95% CI, 51.86-80.24), and a specificity value of 96.29% (95% CI, 94.65-97.52).

In Figure 3, rater 1 (R1) and rater 2 (R2) have a little higher PEA values than those of AI, with no difference in PCA values. The kappa of R1 is just higher than the AI, which is on the level of R2 and R3. Overall, the metrics for Doppler show higher agreement than with SH.

**Figure 4:**
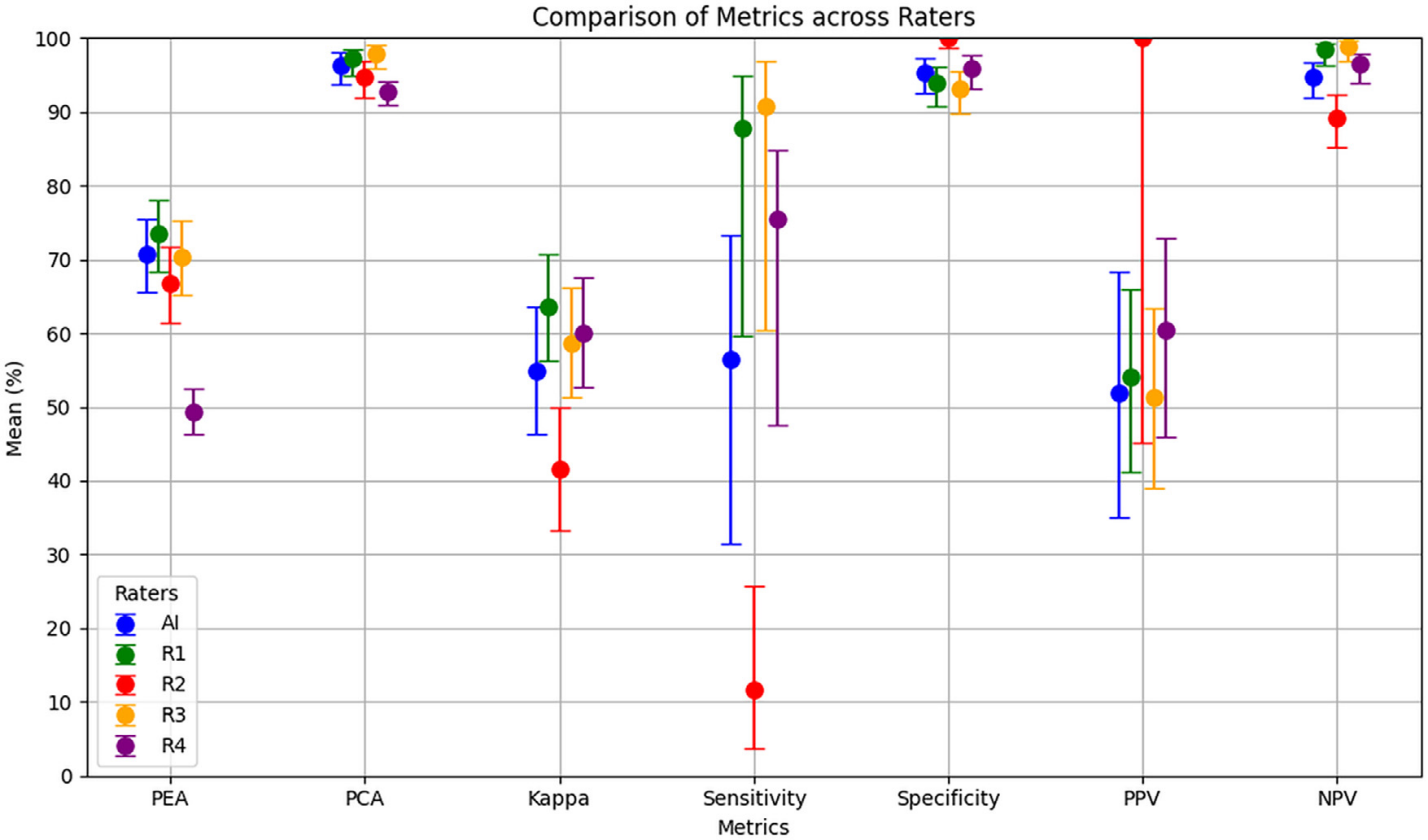
Osteophyte grading agreement. Osteophyte grading agreement of the AI vs all human raters is shown in blue. The performance of individual human raters vs the other human raters is shown as follows: rater 1 (R1) in green, rater 2 (R2) in red, rater 3 (R3) in yellow, and rater 4 (R4) in purple. Mean values and 95% CIs are visible in the plot. NPV, negative predictive value; PCA, percent close agreement; PEA, percent exact agreement; PPV, positive predictive value.

The agreement of the AI model to grade osteophytes compared with the 4 experts is shown in Figure 4. Osteophyte grading agreement of the AI vs human raters showed a kappa value of 0.56 (95% CI, 0.48-0.64), a PEA value of 70.69% (95% CI, 65.57-75.45), a PCA value of 96.28% (95% CI, 93.70-98.01), a sensitivity value of 56.43% (95% CI, 31.56-73.36), and a specificity value of 95.36% (95% CI, 92.44-97.36). In Figure 4, it can be seen that while AI, R1, R2, and R3 gradings are very similar in agreement, R4 grading is lower in PEA value. The distribution of disease grades by the AI and the expert consensus is shown in Table 1. The expert consensus, defined by the majority rating of the rheumatologists, served as the reference standard for all binary performance metrics.

For the AI model test (with agreement metrics of the trained segmentation network on the test data), the values for precision and recall are close for all tissue types (see Supplementary Table S3), suggesting that the segmentation errors are equally distributed as false positives and false negatives. The agreement in table form of the AI model against all human raters on the test data set, and each rater against all other human raters, can be seen in Table 2.

**Table 2:** Performance metrics of the artificial intelligence (AI) model vs human experts

Pathology/rater	Kappa^a^ (95% CI)	PEA (%, 95% CI)	PCA (%, 95% CI)	Sensitivity (%, 95% CI)	Specificity (%, 95% CI)
SH					
AI vs human raters	39.16 (34.5-43.8)	51.77 (48.8-54.7)	91.03 (89.2-92.6)	46.19 (39.1-53.3)	90.43 (88.4-92.3)
Rater 1 vs other human raters	45.74 (41.0-50.5)	55.25 (52.3-58.2)	93.87 (92.3-95.2)	46.01 (38.9-53.2)	91.99 (90.0-93.7)
Rater 2 vs other human raters	47.38 (42.8- 51.9)	56.30 (53.3-59.3)	94.20 (92.7-95.5)	54.23 (46.9-61.4)	91.13 (89.1-92.9)
Rater 3 vs other human raters	36.42 (31.6- 41.3)	48.30 (45.3-51.3)	90.03 (88.1-91.8)	34.47 (28.0-41.4)	95.45 (93.8-96.7)
Rater 4 vs other human raters	43.73 (39.2- 48.3)	49.34 (46.3-52.4)	92.67 (91.0-94.1)	71.04 (63.7-77.5)	80.84 (78.1-83.4)
Rater 5 vs other human raters	38.81 (33.6-44.0)	49.71 (46.5-52.9)	91.37 (89.4-93.0)	54.49 (46.3-62.5)	84.07 (81.4-86.5)
Doppler activity					
AI vs human raters	60.64 (53.9- 67.4)	80.49 (77.5-83.2)	97.13 (95.7-98.2)	67.31 (51.9-80.2)	96.29 (94.7-97.5)
Rater 1 vs other human raters	74.12 (68.0-80.3)	87.34 (84.7-89.7)	98.81 (97.7-99.5)	86.67 (71.7-94.8)	97.84 (96.4-98.8)
Rater 2 vs other human raters	70.21 (63.5- 76.9)	84.20 (82.7-85.5)	98.56 (98.0-98.9)	79.75 (64.2, 90.1)	97.83 (96.5- 98.8)
Rater 3 vs other human raters	68.82 (62.9- 74.7)	86.88 (85.6-88.0)	98.74 (98.2-99.0)	61.13 (45.6- 75.1)	99.55 (98.7-99.9)
Osteophytes					
AI vs human raters	55.98 (48.4- 63.6)	70.69 (65.6-75.5)	96.28 (93.7-98.0)	56.43 (31.6-73.4)	95.36 (92.4-97.4)
Rater 1 vs other human raters	63.50 (56.3- 70.7)	73.40 (68.3- 78.1)	97.23 (95.0- 98.5)	87.74 (59.7- 94.9)	93.85 (90.7- 96.1)
Rater 2 vs other human raters	41.58 (33.2- 50.0)	66.73 (61.5- 71.7)	94.78 (91.9- 96.9)	11.68 (3.8- 25.6)	100.0 (98.8- 100.0)
Rater 3 vs other human raters	58.71 (51.3-66.1)	70.39 (65.2-75.2)	97.93 (95.9-99.1)	90.78 (60.4-96.9)	93.12 (89.9-95.5)
Rater 4 vs other human raters	60.11 (52.7-67.6)	49.34 (46.3-52.4)	92.67 (91.0-94.1)	75.48 (47.4-84.9)	95.85 (93.1-97.6)

Values represent the mean performance metric with the 95% confidence interval provided in parentheses. PCA, percent close agreement (score match within ± 1 unit); PEA, percent exact agreement (exact score match); SH, synovial hypertrophy.

Additional performance metrics, including positive predictive value (PPV) and negative predictive value (NPV), are available in Supplementary Table S4.

a To ensure a consistent scale across all metrics, kappa values have been multiplied by 100. The table displays the agreement for the AI vs human raters in bold, followed by each individual human rater compared with the other human raters.

## DISCUSSION

AI in image processing in rheumatology is a rapidly developing field [22]. Several previous models, including our own, have focused on assessing single features of 1disease, such as osteophytes in hand OA [13,14,23]. We presented performance data for a multifeature AI model that assesses several hallmarks of the most prevalent joint diseases, including both degenerative changes (osteophytes) and inflammatory activity (SH and Doppler). Learning the complex interplay between these features is essential for making AI a more usable tool for clinicians in the diagnostic process of patients with joint pain.

The AI model demonstrated agreement metrics for SH and osteophyte grading that were within the range of interrater variability among experienced experts, suggesting that its scoring reproducibility is promising. Nonetheless, the model was not designed to filter low-quality images, and moderate performance for SH highlights the importance of continued refinement. In SH grading, the PEA agreement overall was lower than for Doppler or osteophyte severity assessment, which is broadly consistent with previous studies.

Reported interrater agreement in the literature varies widely: for SH, PEA ranges from 47.5% to 76.0%, PCA from 86.7% to 100.0%, and kappa from 0.25 to 0.78 [16,24,25]. These values depend heavily on methodological factors, such as whether real-time or static image scoring was used, and whether prestudy alignment or consensus calibration was conducted. Because our study involved only static images without prealignment, our results likely reflect a more pragmatic, real-world scenario of uncalibrated image-based scoring. Direct comparison with earlier reliability exercises based on real-time scanning should therefore be made cautiously.

In this study, the number of expert raters varied between pathology domains (3-5 raters). Although large-scale studies aimed at developing international consensus often involve larger panels (eg, 10-12 experts) [8,16], a reference panel of 2 to 5 experts is common and well-accepted in studies validating AI models against human expert interpretation [13,14]. For example, Wu et al [13] utilised experts to establish a consensus reference, and Fiorentino et al [14] utilised a single expert for ground-truth generation. The robustness of our reference standard is supported by our sensitivity analysis for SH, which demonstrated that reducing the rater pool to the 3 experts who completed all scores did not significantly alter the agreement metrics. This indicates that a panel of 3 to 5 experts provided a stable and valid baseline for the primary evaluation and that the observed variability is likely a reflection of the inherent complexity of the scoring system rather than the number of participants.

The AI model was neither the highest performer nor the lowest performer for any of the metrics. Like human experts, the model finds scoring higher degrees of pathology harder than identifying healthy joints, as indicated by high specificity values and lower sensitivity values. Although ultrasound experts are often better at distinguishing artefacts and noise from true Doppler signals, the AI model provides a perfectly reproducible and deterministic outcome. By consistently generating identical scores for the same input data, the use of such a model could eliminate the intrarater and intercentre variability that often challenges multicentre clinical trials.

The use of this AI model could be implemented in servers collecting images from scanners in the clinic, directly in the ultrasound systems, or in fully automated ultrasound systems [18]. This could allow for real-time analysis with consistent quality approval. In time, with an increasing number of training images and rigorous quality assessment, such a model could become a standard for assessing disease activity on joint ultrasound images.

Several limitations should be considered when interpreting our findings. First, although our results demonstrate the AI model’s potential in hand OA, it is important to note that ultrasound-detected osteophytes alone are not sufficient to diagnose clinical hand OA according to ACR criteria [9]. Osteophytes are common in asymptomatic individuals, especially with age, and are not necessarily indicative of disease. Currently, no international hand OA definition includes ultrasound-based osteophyte grading. Furthermore, the current model does not discriminate between joint effusion and SH, which poses a risk of overestimating synovitis in joints where effusion is caused by hand OA.

Second, the anatomical coverage was not uniform across all pathology domains. SH and Doppler activity were not evaluated in DIP joints, and osteophytes were not evaluated in the wrist (RCIC) joints. Consequently, the model’s performance for these specific joint-pathology combinations remains untested in the present data set. Additionally, the study exclusively used dorsal midline views. Although this follows EULAR–OMERACT recommendations, it may reduce sensitivity compared with multiview assessments (eg, volar or lateral scans) [16]. However, since both the AI and experts evaluated the same images, this technique does not affect the comparability of their scores.

Third, the study intentionally did not include reader alignment or consensus calibration. Although this approach was taken to simulate a more realistic clinical scenario, the lack of a formal calibration phase likely contributed to the modest interrater agreement observed for SH. Furthermore, the model currently utilises a single segmentation mask for both hyaline cartilage and SH [14,23]. Although the classification algorithm is trained on expert-graded images to minimise the impact of this overlap, the visual separation of these structures remains a point for future refinement.

Finally, the relatively low prevalence of high-grade pathology in our data set likely reduced achievable kappa values and limited evaluation across the full disease spectrum. Due to the voluntary nature of the study, not all raters completed every domain. Although sensitivity analyses confirmed the robustness of the SH results, a larger, fully overlapping panel of experts would be ideal for future validation. Future studies should integrate clinical and laboratory data, refine anatomical segmentation, and adjust for intrapatient correlations.

This study demonstrated that an AI model can assess synovial hypertrophy, Doppler activity, and osteophytes in the small joints of the hand with agreement levels comparable with those of experienced sonographers. By providing deterministic and standardised grading, the model addresses the inherent challenge of interrater variability in ultrasound assessment. There are several opportunities for further development, which are currently being addressed. This information is particularly relevant for the visual separation of cartilage and synovial tissue in the segmentation masks. Overall, these findings support the potential of AI as a scalable tool for standardised joint assessment in both clinical practice and research. Future validation in broader clinical settings should integrate clinical context and real-time workflows to further strengthen the clinical utility of hand OA and inflammatory arthritis.

## Competing interests

SAJ is supported by a grant from the Region of Southern Denmark (21/17499). SAJ and TRS report a relationship with ROPCA Aps that includes equity or stocks. ABHW is a full-time employee of ROPCA Aps.

## Appendix Supplementary materials

Image, application 1

Image, application 2

## Footnotes

Supplementary material associated with this article can be found in the online version at doi:10.1016/j.ero.2026.01.015.
