# Supplementary material for: Performance of an artificial intelligence model compared with multiple human experts in scoring synovitis and osteophyte severity on joint ultrasound images
Source: EULAR Rheumatol Open. 2026 Mar 2;2(1):274–82. doi: 10.1016/j.ero.2026.01.015 (PMC13292146; doi:10.1016/j.ero.2026.01.015)
Supplement: Supplementary file 2 [file mmc2.docx]

**Supplementary Material**

**Design of AI model**

The segmentation model which segments the ultrasound images is a Convolutional Neural Network (CNN) built on the UNet++ architecture ^1^. UNet++ expands upon this architecture and adds connections from the encoder to the decoder in the network for more precise segmentation results. UNet++ also has several output branches, which makes it possible to make tradeoffs between precision and latency.

The segmentation model was trained for 300 epochs with the following hyperparameters: network depth: 5, convolutional filters: 32 to 512, Dice loss function, Adam optimization, learning rate 1e-4, fixed dropout rate of 0.1, applying data augmentation with a probability of 20%, and had a total of 9,041,733 trainable parameters. The performance of the segmentation network (Supplementary Table S3) to segment synovia, bones, osteophytes, tendons and artefacts was tested on 716 images which were not part of the training- and validation processes.

**Definitions of SH, Doppler activity and osteophyte**

Synovial hypertrophy (SH) is defined as the presence of abnormal hypoechoic synovial tissue within the capsule that is not displaceable and poorly compressible and that may exhibit Doppler signals.^2^ Presence of Doppler signal within synovial hypertrophy, indicating active inflammation.

Osteoarthritis (OA) osteophyte is defined as a step-up bony prominence at the bony margin that is visible in 2 perpendicular planes.^2^

**Supplementary Table S1: Grading system for synovial hypertrophy, Doppler activity, and osteophytes**

| **Grade** | **SH explanation** | **Doppler explanation** | **Osteophyte explanation** |
| --- | --- | --- | --- |
| Grade 0 | No SH | No Doppler activity | No osteophytes |
| Grade 1 | SH up to the level of the horizontal joint line | Up to three single Doppler spots or up to one confluent spot and two single spots or up to two confluent spots | One osteophyte with no or marginal vertical displacement from the bone |
| Grade 2 | SH extending beyond the joint line and with a concave or flat upper surface | Greater than grade 1 but less than 50% Doppler signals within the synovia | One osteophyte with vertical displacement from the bone or more than one osteophyte where all have no or marginal vertical displacement from the bone |
| Grade 3 | SH extending beyond the joint line and with a convex surface | Greater than grade 2 (more than 50% Doppler signals within the synovia) | More than one osteophyte where at least one has a vertical displacement from the bone |

**Supplementary Table 1**: Grading system for synovial hypertrophy, Doppler activity and osteophytes

**Supplementary Table S2: Distribution of ultrasound images and videos used for AI training, validation, and testing**

|  | **Segmentation model** | | | **Disease grading** | | |
| --- | --- | --- | --- | --- | --- | --- |
| Joint Type | Training Set | Validation Set | Test Set | Synovial hypertrophy Test Set | Doppler  Test Set | Osteophyte  Test Set |
| MCP | 2123 | 296 | 299 | 526 | 369 | 127 |
| PIP | 2102 | 258 | 250 | 507 | 386 | 111 |
| DIP | 1095 | 79 | 99 | 0 | 0 | 113 |
| Wrist RCIC | 563 | 82 | 68 | 247 | 85 | 0 |
| **Total** | **5883** | **715** | **716** | **1280** | **840** | **351** |

**Supplementary Table 2**: Number of joint images used in training the AI model.

**Supplementary Table S3: Performance metrics of the trained AI segmentation network**

| **Tissue type** | **IOU** | **Precision (%)** | **Recall (%)** | **FDR (%)** | **FNR (%)** |
| --- | --- | --- | --- | --- | --- |
| Synovium | 0.55 | 68.54 | 69.00 | 31.46 | 31.00 |
| Bone | 0.86 | 93.57 | 91.22 | 6.43 | 8.78 |
| Osteophyte | 0.29 | 35.71 | 34.31 | 64.29 | 65.69 |
| Tendon | 0.59 | 71.96 | 71.58 | 28.04 | 28.42 |
| Artifact | 0.62 | 69.20 | 69.85 | 30.80 | 30.15 |

**Supplementary Table 3:** Performance of the trained segmentation network on the test data. IOU = Intersection Over Union, FDR = False Discovery Rate, FNR = False Negative Rate. Precision is also known as positive predictive value (PPV), and recall is also known as sensitivity.

**Supplementary Table S4: Expanded performance metrics (PPV and NPV) for the AI model and human raters**

| Pathology / Rater | Positive Predictive Value (PPV) (%, 95% CI) | Negative Predictive Value (NPV) (%, 95% CI) |
| --- | --- | --- |
| Synovial Hypertrophy (SH) | | |
| AI versus human raters | 51.50 (44.15, 58.78) | 87.47 (85.25, 89.45) |
| Rater 1 versus other human raters | 56.67 (48.91, 64.18) | 87.02 (84.76, 89.05) |
| Rater 2 versus other human raters | 57.13 (49.99, 64.03) | 88.90 (86.73, 90.80) |
| Rater 3 versus other human raters | 64.56 (55.15, 73.17) | 85.07 (82.67, 87.25) |
| Rater 4 versus other human raters | 41.33 (35.60, 47.24) | 92.98 (91.00, 94.62) |
| Rater 5 versus other human raters | 40.55 (34.10, 47.26) | 89.51 (87.17, 91.54) |
| Doppler Activity | | |
| AI versus human raters | 55.65 (42.36, 68.20) | 97.70 (96.33, 98.66) |
| Rater 1 versus other human raters | 68.56 (53.29, 81.38) | 99.18 (98.20, 99.68) |
| Rater 2 versus other human raters | 68.57 (53.81, 80.80) | 98.68 (97.54, 99.36) |
| Rater 3 versus other human raters | 90.42 (74.47, 97.83) | 97.35 (95.85, 98.42) |
| Osteophytes | | |
| AI versus human raters | 51.88 (35.07, 68.33) | 94.76 (91.85, 96.74) |
| Rater 1 versus other human raters | 53.97 (41.27, 65.97) | 98.40 (96.33, 99.35) |
| Rater 2 versus other human raters | 100.0 (45.13, 100.0) | 89.12 (85.29, 92.24) |
| Rater 3 versus other human raters | 51.24 (38.93, 63.33) | 98.85 (96.92, 99.62) |
| Rater 4 versus other human raters | 60.46 (45.86, 72.99) | 96.46 (93.95, 97.96) |

**SupplementaryTable 4:** Additional performance metrics (PPV and NPV) for the AI Model and individual human raters.

**Supplementary Table S5: Sensitivity analysis of the AI Model performance against human raters 1–3.**

| **SH** | **Rater** | **Kappa* (+/- 95% CI)** | **PEA (%, +/- 95% CI)** | **PCA (%, +/- 95% CI)** | **Sensitivity (%, +/- 95% CI)** | **Specificity (%, +/- 95% CI)** | **PPV (%, +/- 95% CI)** | **NPV (%, +/- 95% CI)** |
| --- | --- | --- | --- | --- | --- | --- | --- | --- |
| AI versus human raters 1-3  Rater 1 versus other human raters  Rater 2 versus other human raters  Rater 3 versus other human raters | | 38.90 (34.23 to 43.57) | 53.82 (50.94 to 56.67) | 91.28 (89.52 to 92.82) | 51.36 (43.49 to 59.18) | 89.65 (87.60 to 91.46) | 45.34 (38.26 to 52.58) | 91.53 (89.64 to 93.18) |
|  |  | 46.77 (42.11, 51.43) | 57.44 (54.52, 60.32) | 94.48 (93.02, 95.71) | 53.90 (45.65, 62.00) | 91.44 (89.51, 93.11) | 50.33 (42.80, 57.87) | 92.35 (90.51, 93.93) |
|  |  | 48.50 (43.98, 53.02) | 58.37 (55.44, 61.25) | 94.54 (93.08, 95.77) | 63.18 (54.67, 71.10) | 89.62 (87.54, 91.45) | 47.10 (40.00, 54.29) | 94.20 (92.52, 95.60) |
|  |  | 39.52 (34.53, 44.51) | 53.51 (50.54, 56.46) | 92.50 (90.79, 93.97) | 39.22 (31.92, 46.89) | 94.93 (93.34, 96.24) | 58.87 (49.43, 67.84) | 89.43 (87.36, 91.26) |

**Supplementary Table S5:** The performance of the AI Model against human raters 1-3 (Test Dataset) and the performance of each rater against all other human raters. *Kappa values are multiplied by 100 to have the same scale for all metrics.

**References**

1. Stoyanov D, Taylor Z, Carneiro G, et al., eds. UNet++: A Nested U-Net Architecture for Medical Image Segmentation. Deep Learning in Medical Image Analysis and Multimodal Learning for Clinical Decision Support; 2018 2018//; Cham. Springer International Publishing.

2. Bruyn GA, Iagnocco A, Naredo E, et al. OMERACT Definitions for Ultrasonographic Pathologies and Elementary Lesions of Rheumatic Disorders 15 Years On. *The Journal of rheumatology* 2019;46(10):1388. doi: 10.3899/jrheum.181095
